# Endurance and Cycle-to-cycle Uniformity Improvement in Tri-Layered CeO_2_/Ti/CeO_2_ Resistive Switching Devices by Changing Top Electrode Material

**DOI:** 10.1038/srep39539

**Published:** 2017-01-12

**Authors:** Anwar Manzoor Rana, Tahira Akbar, Muhammad Ismail, Ejaz Ahmad, Fayyaz Hussain, Ijaz Talib, Muhammad Imran, Khalid Mehmood, Khalid Iqbal, M. Younus Nadeem

**Affiliations:** 1 Thin Films Laboratory, Department of Physics, Bahauddin Zakariya University, Multan-60800, Pakistan; 2 Department of Physics, Government College University Faisalabad, Layyah Campus Layyah-31200, Pakistan; 3 Institute of Chemical Sciences, Bahauddin Zakariya University, Multan-60800, Pakistan; 4 Department of Radiation Oncology, Shaukat Khanum Memorial Cancer Hospital and Research Centre, Lahore-54000, Pakistan

## Abstract

Resistance switching characteristics of CeO_2_/Ti/CeO_2_ tri-layered films sandwiched between Pt bottom electrode and two different top electrodes (Ti and TaN) with different work functions have been investigated. RRAM memory cells composed of TaN/CeO_2_/Ti/CeO_2_/Pt reveal better resistive switching performance instead of Ti/CeO_2_/Ti/CeO_2_/Pt memory stacks. As compared to the Ti/CeO_2_ interface, much better ability of TaN/CeO_2_ interface to store and exchange plays a key role in the RS performance improvement, including lower forming/SET voltages, large memory window (~10^2^) and no significant data degradation during endurance test of >10^4^ switching cycles. The formation of TaON thinner interfacial layer between TaN TE and CeO_2_ film is found to be accountable for improved resistance switching behavior. Partial charge density of states is analyzed using density functional theory. It is found that the conductive filaments formed in CeO_2_ based devices is assisted by interstitial Ti dopant. Better stability and reproducibility in cycle-to-cycle (C2C) resistance distribution and V_set_/V_reset_ uniformity were achieved due to the modulation of current conduction mechanism from Ohmic in low field region to Schottky emission in high field region.

Numerous technologies are being used for the advancement of non-volatile memory (NVM) to replace the existing conventional memory devices^1^,^2^. Resistive random access memory (RRAM) has the inordinate prospective to be the subsequent generation memory device. RRAM has attracted increasing attention due to its superior features; including simple structure, high resistance ratio, low operating voltages, long data retention time, high density, excellent scalability and compatibility with the standard complementary metal oxide semiconductor (CMOS) memory devices^3^,^4^. It is based on the principle of resistance variation effect of oxide material between two (or greater than two) resistance states (“0” and/or “1”) which can be switched alternatively within nanoseconds^1^,^5^.

Several research groups are studying different materials such as rare earth oxides, polymers, transition metal oxides and biomaterials as insulator to develop NVM devices^6^. But rare-earth oxide materials have proved themselves superior by representing better resistive switching (RS) characteristics for the development of NVM devices^7^,^8^. One of the rare-earth metal oxides is CeO_2_, which makes use of the oxygen getting ability of Ce. As it can go through repeatable redox (Ce^+3^/Ce^+4^) cycles based on reduction of cerium from Ce^+4^ to Ce^+3^. Importance of CeO_2_ is not only due to accepting/discharging oxygen ions but also due to the valency change between Ce^+4^ to Ce^+3^ states^9^,^10^,^11^.

Doping of metallic materials into the metal oxides can also enhance the switching characteristics of RRAM device^12^. Oxygen vacancies have the ability to form clusters around the metal dopants, in this way switching process can be controlled by construction and destruction of vacancy-based filaments. But still it is difficult to comprehend whether metal dopants are directly involved in vacancy formation or support the formation process^13^. In addition, the metal oxide (insulator) and top electrode has ample impact on upgrading the performance of RRAM devices^14^. Further study is required to control the impact of top electrodes on RS behavior of such RRAM devices.

Exploring the role of electrode metals on the resistive switching properties of RRAM devices provides not only essential information to understand the underlying switching mechanism of the devices, but also useful guidelines for the optimization of the switching performance. The selection of electrode material depends on the type of contact (Ohmic, Schottky etc.) it makes with the oxide layer, so the work function and electronegativity of metals are important. In addition, metals which can reduce the formation energy of oxygen vacancies in oxide play key role in resistive switching process. Choice of electrode metals determines the mobile species in the oxide layer of the devices^15^. The electrodes which are capable of reserving oxygen ions, or capable of inducing the migration of vacancies inside the oxide layer, can lead to superior bipolar resistive switching behaviors^16^,^17^,^18^. The band alignment between the switching oxide layer and the metal electrodes also controls the interface resistance, which in turn influences the current level, switching power and conduction mechanism of the devices^18^. A number of physical mechanisms have been proposed to explain resistive switching behavior of RRAM devices^19^. In an asymmetric metal-insulator-metal (MIM) structure where a Schottky contact and an Ohmic contact are formed at the two interfaces, it is generally believed that Schottky interface dominates the bipolar resistive switching behavior^20^. However, the electrical characterizations which can reveal the dynamic change of the depletion layer has rarely been reported, and the switching non-volatility mechanism in oxides is unclear. Besides, little attention has been paid to the voltage polarity for the high resistance state (HRS) and low resistance state (LRS). Until now, the electrode material dependence on the resistive switching properties of Ti-doped CeO_2_ thin films has not been reported.

In the present work, the influence of two different Ohmic top electrodes (TaN and Ti) on the resistive switching properties of tri-layered CeO_2_/Ti/CeO_2_ films is investigated. Due to the formation of thinner interfacial TaON layer, the device using TaN as top electrode (TE) shows consistently higher *R*_OFF_/*R*_ON_ (>10^3^) than the one with Ti electrode under the same bias conditions. Based on the density function theory calculations and current transport characteristics during the switching phenomenon, switching mechanism and the physical origin of non-volatility in Ti-incorporated CeO_2_ based MIM RRAM structures have been discussed in detail as it is expected that ultrathin Ti layer may act as a defect distribution regulator. The present results have also been compared with the already existing theories/experiments.

## Results and Discussion

### Structural Analyses

**Figure 1:**
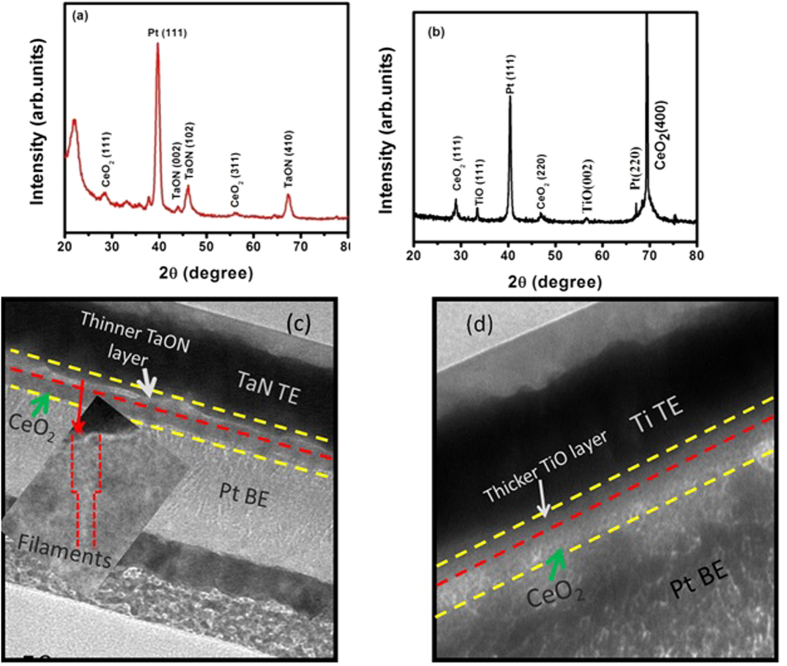
X-ray diffraction patterns for the (**a**) TaN/CeO_2_/Ti/CeO_2_/Pt, (**b**) TaN/CeO_2_/Ti/CeO_2_/Pt stacks and Cross-sectional HRTEM images of (**c**) TaN/CeO_2_/Ti/CeO_2_/Pt, (d) Ti/CeO_2_/Ti/CeO_2_/Pt stacks.

X-ray diffraction (XRD) analysis was performed after studying the resistive switching characteristics of each device. XRD spectra were obtained by using grazing incidence (3°) on the TaN/CeO_2_/Ti/CeO_2_/Pt and Ti/CeO_2_/Ti/CeO_2_/Pt stacks as shown in Fig. 1(a,b). Both TaN and Ti TE-based structures show some broad peaks at diffraction angles of 28.5°, 47.5° and 69.4° which are related to CeO_2_ (111), (220), (400) reflections respectively, according to JCPDS Card No. 34-0394 indicating weak polycrystalline fluorite cubic structure. Figure 1(a) depicts three weak XRD reflections analogous to (002), (102) and (410) planes of tantalum oxynitride (TaON) hexagonal structure according to JCPDS Card No. 72-2067, indicating the formation of interfacial layer close to top electrode. In addition, the presence of (111) and (002) peaks representing the reflections of TiO (JCPDS # 82-0803) in the XRD pattern of Ti TE-base device might lead to the formation of a thicker interfacial layer between Ti and CeO_2_ interface. While XRD pattern of TaN TE device illustrates no reflection related to the presence of Ti as element and/or to any TiO interlayer sandwiched between top and bottom CeO_2_ films.

Being more oxidizable as compared to TaN, Ti extracts more oxygen ions to form TiO thicker interfacial layer as compared to a thinner TaON layer during repeated switching process. So it is expected that the thinner TaON interfacial layer might play crucial role in the improvement of resistive switching characteristics. Cross-sectional view of the TaN TE base device through HRTEM image (Fig. 1(c)) clearly illustrates the formation of thinner TaON interfacial film. However, HRTEM image shown in Fig. 1(d) noticeably reveals that thicker interfacial layer of TiO is formed at the top region of Ti/CeO_2_ interface. XRD and HRTEM analyses of Ti TE-base device confirm that Ti extracts more oxygen ions and generates more oxygen vacancies in the upper CeO_2_ layer. In addition, owing to the strong oxygen affinity of Ti metal, oxygen ions from the upper CeO_2_ layer tend to migrate toward the interface between Ti and CeO_2_, resulting in the formation of a thicker TiO interfacial layer^26^. Furthermore, the HRTEM and XRD patterns illustrate that microstructure of CeO_2_ is weak polycrystalline, which is insensitive to the buried Ti layer in both devices. But there is an obvious interfacial layer either TaON or TiO formed at the interface of upper CeO_2_ and TE. Cross-sectional view of the TaN TE base device through HRTEM image (Fig. 1(c)) clearly illustrates the formation of thinner TaON interfacial film.

**Figure 2:**
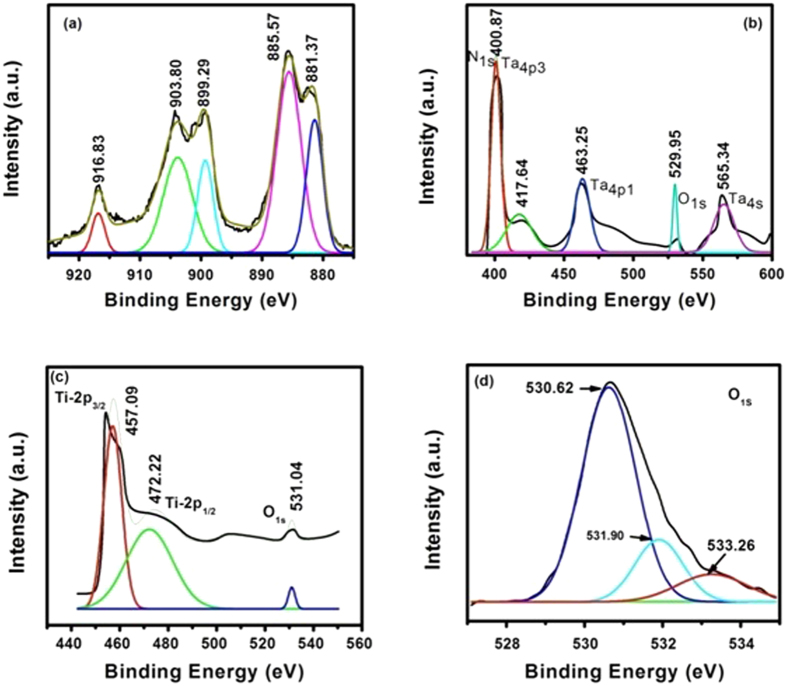
XPS binding energy profiles. (**a**) CeO_2_ layers, (**b**) TaON profile for TaN/CeO_2_/Ti/ CeO_2_/Pt devices, (**c**) Trace profile of Ti in Ti/CeO_2_/Ti/CeO_2_/Pt devices and (**d**) O 1 s profile in Ti/CeO_2_/Ti/CeO_2_/Pt devices.

For further characterization, we conducted XPS analyses to investigate the surface composition and chemical state of the M/CeO_2_/Ti/CeO_2_/Pt devices. Figure 2(a) shows Ce 3d XPS spectrum along with fitted deconvolutions of M/CeO_2_/Ti/CeO_2_/Pt devices. Peaks located at 916.83, 903.80, and 899.29 eV are attributed to Ce^3+^ 3d_3/2_ final states, indicating that the main valence of cerium in the sample is Ce^3+^ (54.9%), regardless of the valences of starting ceria^27^. Furthermore, two strong peaks at 885.57 and 881.37 eV assigned to Ce 3d_5/2_ for Ce^4+^ final states indicate a handful of Ce^4+^ ions (45.1%) existed in the devices^28^. Therefore, it can be reasonably concluded that there are sufficient number of defects existing in the device reflecting the concentration of oxygen vacancies. Figure 2(b) shows the XPS trace spectrum in which O 1 s energy state at about 530 eV corresponds to TaON due to nonlattice oxygens^29^. While, Ta 4p_3/2_ peak shows slightly asymmetric shape and binding energy of this peak has been shifted to higher energy as compared to its value for metallic Ta 4p_3/2_ (~404 eV) supporting the formation of TaON because of the larger electronegativity of oxygen^30^. Since during forming process sufficiently large applied bias field attracts nonlattice oxygen ions in the CeO_2_ layer toward TaN electrode. Consequently, large number of oxygen vacancies are accumulated in the oxide/TaN interface, which can modulate the conductivity of CeO_2_, resulting in the device to switch from HRS to LRS. During negative biasing, oxygen ions are released back from the TaN electrode and annihilate the oxygen vacancies rupturing the filaments. Thus, resistive switching mechanism involves the physisorption or chemisorption of oxygen ions at TaN/CeO_2_ interface for different bias polarities. Figure 2(c) shows the high-resolution Ti 2p XPS spectrum for the Ti/CeO_2_/Ti/CeO_2_/Pt devices. The Ti 2p_1/2_ and Ti 2p_3/2_ peaks are observed at the binding energies of 472.22 eV and 457.09 eV, respectively with a difference of about 15.13 eV inconsistent with Ti 4þ oxidation states of TiO_2_ and may lead to TiO or TiO_2−x_^31^. Further support is provided by O 1 s XPS spectrum shown in Fig. 2(d), which depicts three peaks corresponding to binding energies of 530.62 eV, 531.9 eV and 533.26 eV, respectively. These peaks are slightly blue shifted as compared to corresponding peaks at 529.59 eV related to the oxygen ions in TiO_2_^32^, and at 531.39 eV and 532.24 eV associated with oxygen vacancies and the oxygen-based molecules adsorbed on the surface of TiO_2_, respectively leading to TiO or TiO_2−x_ states^33^. These XPS results demonstrate the presence of oxygen vacancies in the Ti/CeO_2_/Ti/CeO_2_/Pt devices, which can act as donors for the charge carriers and trap sites of electrons^34^.

### Current–Voltage Characteristics

**Figure 3:**
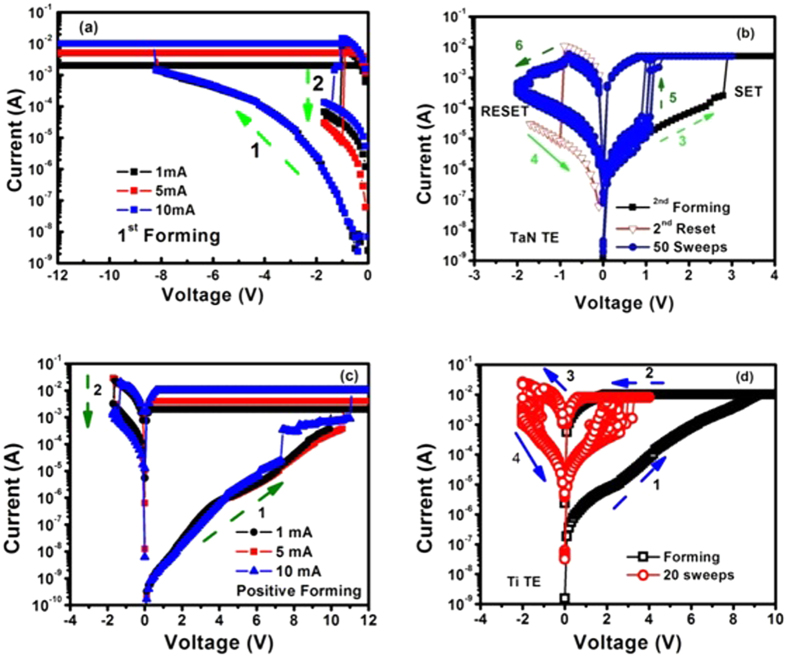
DC sweep I-V curves depicting the double forming phenomenon occurring in the same cell of the TaN/CeO_2_/Ti/CeO_2_/Pt stack. (**a**) First forming (negatively biased) and reset process along with (**b**) Second forming (positively biased) and Reset process. (**c**) I-V curves illustrating only the positively biased electroforming process in TaN/CeO_2_/Ti/CeO_2_/Pt device, (**d**) electroforming and RS behavior of Ti/CeO_2_/Ti/CeO_2_/Pt stacks.

Activation phenomenon for switching the device from high resistance state (HRS) to the low resistance state (LRS) occurring for the first time at relatively high fields is generally called “Electroforming”. It is usually an essential effort for activating the memory devices. In this regards, bias voltages in the range from −12 V to +12 V were applied to both the devices to initiate the switching transition. Moreover, different compliance currents (such as 1, 5 and 10 mA) were used for TaN TE base memory devices, as shown in Fig. 3(a,c), whereas current compliance of only 10 mA is set for Ti TE base devices (Fig. 3(d)). The TaN/CeO_2_/Ti/CeO_2_/Pt stack reveals the double forming process as obvious from Fig. 3(a,b). To trigger the TaN TE-base device through negative biasing, first electroforming, for the pristine device is achieved at voltages (V_form_) ranging from −8 V to −8.2 V, which is associated with the requirement of inducing the conductive paths in the pristine device^35^. A tight distribution of the V_form_ for the devices with TaN electrode is observed in Fig. 3(a) with negative forming (NF). During the NF process, TaN TE attracts the positively charged oxygen vacancies toward the interface, which creates defect states in the depletion layer and reduces the Schottky barrier height and possibly width, resulting in the Ohmic transport and LRS. Subsequently, ‘reset’ process could also be seen when sweeping the voltage back with same negative bias (oxygen ions are repelled from the TaN/CeO_2_ interface that shift the oxygen vacancies away from the filament, and hence restores the Schottky barrier and HRS); this is a unipolar RS-like behavior that converts the device from LRS back to HRS at about −1.0 V of the same negative biasing (Fig. 3(a)).

After completing the first cycle, when negative bias is applied again to TE, no obvious set process (results are not shown here) is noticed after first reset, a permanent hard break down occurred on it. On the other hand, the same memory cell shows successful resistance switching to LRS when a positive voltage is applied to TE of the device, the phenomenon hereby called ‘second forming’ process as shown in Fig. 3(b). As a result, reset process occurs with negative biasing. Successful bipolar RS characteristics with 50 DC cycles (voltage sweeps) were performed to check the uniformity and reliability in the performance as shown in Fig. 3(b) by arrows: 5→6 (blue color plots). During these cycles, TaN TE-base devices reveal reproducible RS characteristics with consistency and uniformity in the RS parameters. For such transitions in the resistance states of the memory device, a sufficient positive bias (V_SET_) is applied and the device transfers from HRS to LRS due to the creation of oxygen vacancies in the upper CeO_2_ film due to drift of oxygen ions to TE by applied electric field. In contrast, an application of negative bias results in a transition from LRS to HRS at certain reset voltage (V_RESET_), it needs oxygen ions to recover CeO_x_ for achieving HRS.

In the case of positive electroforming (PF) only, on applying a positive fixed biasing of ~12 V along with three different current compliances (CC) of 1, 5 and 10 mA to TaN TE as shown in Fig. 3(c), the device transitioned from HRS to LRS at certain voltages ranging from ~9 V to ~10.6 V with increasing CC. This electroforming process has made the devices capable to start RS. So when negative bias voltage is applied to the top electrode in the LRS mode, resistive switching of the devices back to the HRS occurs in an abnormal way (very weak transition) as obvious from Fig. 3(c). This transition represents the reset process (device switched into the “off state”); however, the current is still small (~1 mA) in this state. So it can be said that the reset transition occurs from one LRS to another LRS. That is why, when positive voltage is applied to TE, device does not show any type of RS behavior. This may be due to the reason that the device has not shifted to HRS during this RESET process representing metallic type behavior. It is expected that all the conducting filaments might have not been ruptured during this transition. Analogous type behavior has recently been observed by Kim *et al*.^36^ in the case of Ni/Si_3_N_4_/n^+^-Si memory devices. They attributed such behavior to the formation of conductive filaments from the nano-polycrystalline Si pathways as of broken Si-N bonds or the formation of metallic conducting bridge induced by TE made up of highly diffusible metallic species.

On the other hand, Ti TE-based RRAM devices display only the bipolar characteristics after positive forming as shown in Fig. 3(d) as compared to the TaN TE-base devices. After the initial forming occurred at 9.2 V, HRS was attained at −2.1 V. The subsequent SET occurred at positive bias of 3.3 V. It can be concluded from aforementioned facts that TaN TE base devices possess lesser V_SET_ and V_RESET_ than those for Ti TE RRAM devices. In addition, it is noticed that the currents increase rapidly which result in a lower and tight distribution of formation voltages in NF mode (−8 to −8.2 V) as compared to those in PF mode (9 to 10.6 V) for TaN TE base devices. This suggests that lower formation voltage can protect device degradation^37^.

During forming process in the TaN/CeO_2_/Ti/CeO_2_ devices, high electric field can possibly decompose CeO_2_ molecules and yield Ce-O bonds resulting in oxygen vacancy generation and hence oxygen vacancy based conductive filaments. This process causes the release of oxygen ions from the conducting channels, which can move from the TaON/CeO_2_ top interface region toward Ti/CeO_2−x_ bottom region under NF mode due to negative biasing of TE relative to the BE. Under PF mode, the oxygen ions will move toward the TaN TE because of its positive polarity. In this case, the oxygen ions will be accumulated at the TaON/CeO_2−x_ top interface, i.e., oxygen-rich CeO_2_ film. In this way, oxygen vacancy filaments can be formed in the TaN/CeO_2_/Ti/CeO_2_ stack. The Ti/CeO_2−x_ bottom region will behave as a conducting layer under PF mode.

### Electrical Endurance

**Figure 4:**
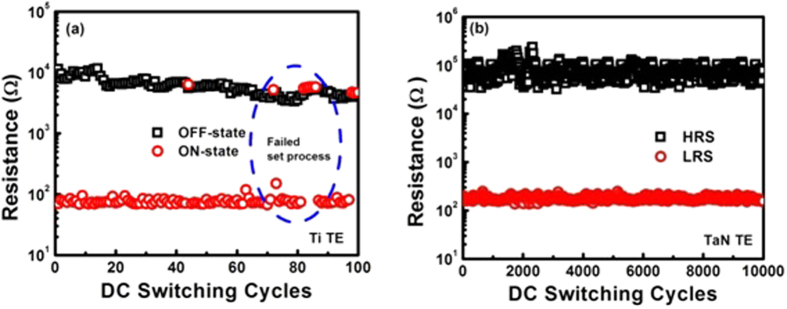
Endurance characteristics against repeated program operations for (**a**) Ti TE (**b**) TaN TE based resistive switching memory devices.

To evaluate the cycle-to-cycle stability and reproducibility of both TaN and Ti TE based devices, their electrical endurance tests were performed as illustrated in Fig. 4(a,b). The current values of both TaN and Ti TE devices were measured at reading voltage of ±0.2 V. These plots clearly illustrate that TaN TE based devices demonstrate a reliable endurance over 10^4^ RS cycles by maintaining a memory window of ~10^2^. Such a wide memory window can fruitfully accomplish the necessity of an efficient memory device for RRAM applications^1^,^2^. Moreover, TaN TE base device shows very stable ON and OFF-state resistances without any observable degradation and are distinctly differentiable. It is noted that LRS shows greater uniformity for more than 10000 switching cycles while HRS is somewhat dispersive. But in the case of Ti TE (Fig. 4(a)) encircled portion showed that SET process has failed during endurance test for less than 100 DC voltage sweeps, because conductive filament size is expected to be increased with repetitive switching cycles.

It is well known that Ti has capability to extract more oxygen ions from the adjacent dielectric layer to form TiO_x_ interlayer thereby creating more vacancies which can further extend or increase the size of CFs^38^,^39^. But this is not observed in our Ti TE based devices perhaps due to similar metals sandwiching the CeO_2_ layer (i.e. Ti/CeO_2_/Ti), since conducting filaments are partially formed. In addition, it is noticed that high bias voltages were required to start RS and rupture the conducting channels between two electrodes and successfully transferring the device into HRS (SET-state). Conducting filaments have ruptured to such an extent that inordinate effort is required to complete the conducting path. The LRS and HRS are getting overlapped at some switching cycles which express the failure of endurance test. The device remains in HRS (RESET-state) for more than one switching cycle exhibiting abortive SET and RESET process.

The current compliance (CC) and SET/RESET voltages are incapable to supply enough number of oxygen ions from the Ti TE to refill the oxygen vacancies and rupture the thicker conducting filaments. So, it may be essential to increase CC or SET/RESET voltages for successful repetitive switching cycles. After 100 DC switching cycles, the density of impurities and/or the size of CFs can be so high that the CF could not be entirely re-oxidized (refilled) anymore (Fig. 4(b)), leading to the failure of switching transitions between SET and RESET process. On the other hand, TaN TE devices reveal successful SET and RESET operations (Fig. 4(b)), which are caused by TaON interfacial layer acting as oxygen ion reservoir in enhancing the oxygen vacancy generation in CeO_2_ layer and subsequent suppression in the randomness of oxygen vacancy filament formation. The TaON interfacial layer might play a vital role in controlling the size of the CFs.

Endurance failure in the Ti/CeO_2_/Ti/CeO_2_/Pt stacks due to a deficiency of oxygen ions for filament re-oxidation may lead to an important conclusion that there should be sufficient number of oxygen ions available for the resistive switching behavior. Chang *et al*.^40^ also reported that failure of the reset process in the Mo/ZnO/Pt structure may be due to a deficiency of oxygen ions for filament re-oxidation. Hence, the existence of oxygen ions in the electrode or in its neighborhood is very important. Such deficiency of oxygen ions in our case can be explained on the basis of the difference in Gibbs free energy of oxide formation by metallic electrodes. The redox of Ti and TaN occurs at the TE/CeO_2_ interface, that is, Ti and TaN oxidize to TiO and TaON respectively, while CeO_2_ is reduced to CeO_2−x_. An oxygen-deficient CeO_2_ layer is formed by the percolation of some type of defects, such as oxygen vacancies, interstitial Ce etc. near the Ti/CeO_2_ and TaN/CeO_2_ interfaces^41^. It is known that higher Gibbs free energy can be associated with more stable interfacial oxide formation, consequently the reset process becomes more difficult^42^,^43^. Such as for Ti TE, Gibbs free energy for TiO_2_ formation is −802.5 kJ/mol as compared to that for TaON formation (−604.96 kJ/mol) in the case of TaN TE base devices. That is why reset process in Ti TE base devices is difficult; on the other hand, lower Gibbs free energy of TaON makes the reset easier for TaN TE base devices resulting in a higher ON/OFF ratio, good endurance and reduction in the set voltages.

### Data Retention Characteristics

**Figure 5:**
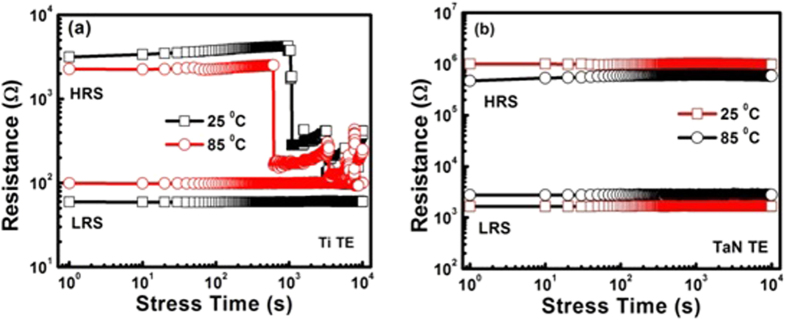
Retention characteristics of the programmed currents for set- and reset-operations as a function of retention time in HRS and LRS at RT and at 85 °C for the (**a**) Ti TE and (**b**) TaN TE based resistive-switching memory devices.

The resistance switching behaviour under an electrical stress is vitally important for the actual application of nonvolatility of RRAM devices. First of all, both TaN and Ti TE base devices were successfully transferred from HRS to LRS (turned ON, SET-state) then electrical stress of +0.2 V was applied for 10^4^ s (with time interval of 10 s) at room temperature and 85 °C. After that the process was repeated in the RESET-state (device turned OFF) for negative electrical stress of −0.2 V applied for 10^4^ s at room temperature and 85 °C. It is noticed that the TaN TE base devices exhibited good stability in the LRS and HRS with no major deterioration during stress time (10^4^ s), confirming the nonvolatility characteristic during room temperature and at 85 °C as obvious from Fig. 5(b). Such improved retention of these devices might be caused by thinner interfacial TaON layer which acts as an oxygen diffusion barrier^44^. However, for Ti TE based devices, results indicate the failure of HRS characteristics after about 10^2^ s during room temperature as well as at 85 °C as shown in Fig. 5(a). In Ti TE based device, thicker interfacial TiO layer caused a failure in the data retention times. It is suggested that HRS characteristics are related to the size of the conductive filaments as explained earlier. In addition, inadequate rupture of too thick conductive filaments during RESET process might also be associated with stronger bonding of Ti-O. Hence thicker TiO interfacial layer is a major factor in the failure of endurance, retention, and wider voltage operation in Ti TE based device.

### Statistical and Cumulative Probability Distributions

**Figure 6:**
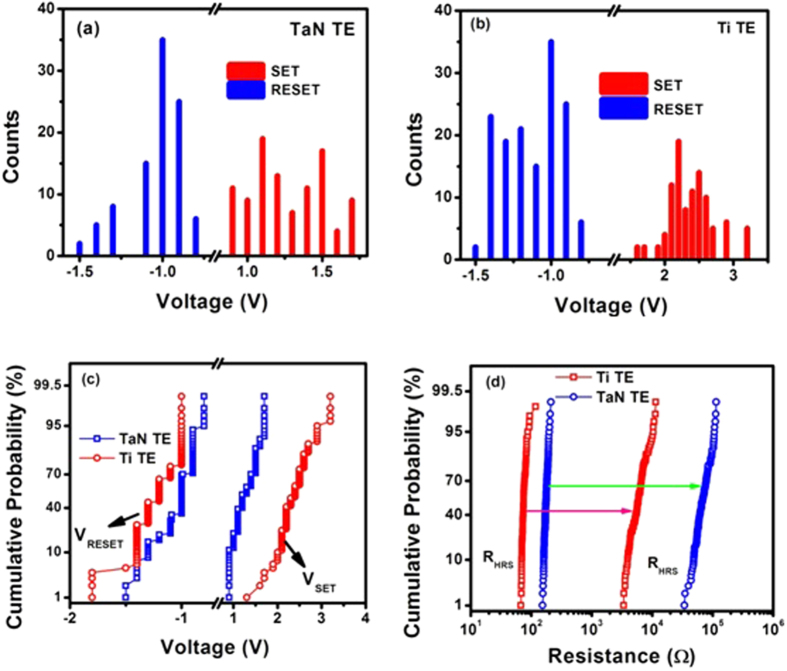
Statistical and cumulative probability distribution of V_set_ and Vre_set_ voltages measured from a device unit for first 100 tests. (**d**) R_LRS_ and R_HRS_ measured under DC voltage sweeps for both TaN and Ti TE based devices.

Excellent uniformity in the operational voltages is still a challenge for the production of RRAM devices^3^,^4^. Results of relative frequency and cumulative probability distribution of V_set_ and V_reset_ voltages for both TaN and Ti TE-base devices replotted for first 100 consecutive switching cycles are shown in Fig. 6(a–c). It can be noted that TaN TE base devices show narrow distribution of V_reset_ (−1.5 to −0.80 V) and V_set_ (0.90 to 1.70 V). But Ti TE devices exhibit relatively wider V_reset_ and V_set_ distributions ranging from −1.8 to −1.0 V and from 1.20 to 3.20 V respectively. The much smaller variations observed in V_set_ and V_reset_ of TaN TE devices lead to better cycle-to-cycle uniformity in their resistive switching properties. The reduction in V_set_ and V_reset_ voltages can be attributed to the high reactivity of TaN with oxygen. The easy formation of the field induced oxygen deficiency in the CeO_2_ film due to the lower formation energy of the TaON^16^ improves the oxygen ion migration during the formation process as explained above.

It may be noted that Ti-doping probably is not able to enhance the local electric field within the CeO_2_ layers. It is worth noting that even the minimum set voltage in Ti TE devices is greater than 1.30 V and also shows a wide dispersion. Such increase of operation voltages may lead to a slight increase of power that the device may consume, but it also means the read voltage can be set higher to avoid outer electrical noise interference. Good cycle-to-cycle uniformity may be attributed to the interfacial effect of the TaON/CeO_x_ interface acting as a large oxygen ion migration barrier and confining the switching in the active oxide^45^.

To compare the memory window and fluctuations in the HRS and LRS resistances in the DC cycles, quantitative estimation has been developed. The Ti TE base devices depict a broad distribution of resistances in HRS and LRS, which is due to the random distribution of the oxygen vacancies in the CeO_2_ film. TaN TE base devices display much better dispersion of both R_HRS_ and R_LRS_. TaN TE may be capable of reducing the electrical insulation of oxide layer; as a result it may decrease the CC level and raise HRS as shown in Fig. 6(d). Here, no overlapping was observed during repeated switching cycles in TaN TE RRAM devices. The TaN TE devices depict large memory window and uniformity as compared to Ti TE RRAM devices. According to the filamentary model of oxygen vacancies or defects^46^, the dispersion of memory switching parameters can be reduced by controlling the filament construction and rupture processes occurring at certain portions near the CeO_x_/TaON interfaces. However, the HRS resistance of the TaN and Ti TE devices during the cycling tests varies significantly, and it can be attributed to the diverse CFs in each switching cycle, which leads to nonuniform RS properties^45^.

### Current Transport Mechanisms

**Figure 7:**
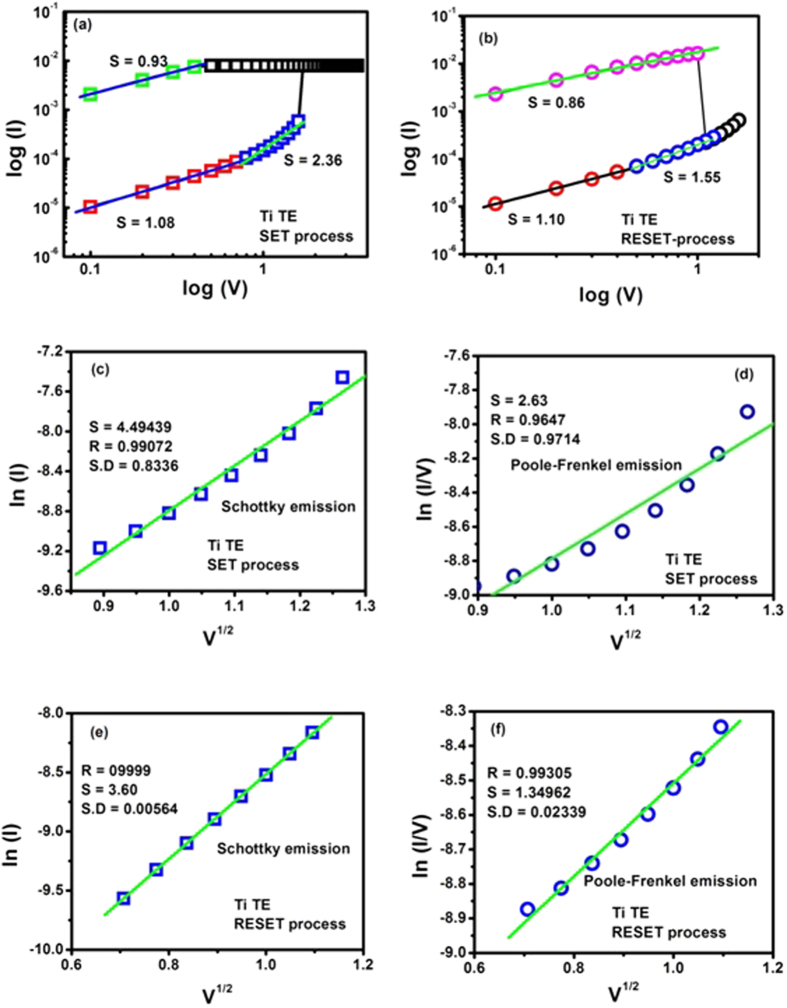
I-V curves fitted in double logarithmic scale for Ti TE memory devices. (**a**) set process (**b**) reset process. I–V curves fitting by Schottky emission (**c**) and Poole–Frenkel emission (**d**) in HRS of positive bias and in HRS of higher negative bias region (**e–f**).

**Figure 8:**
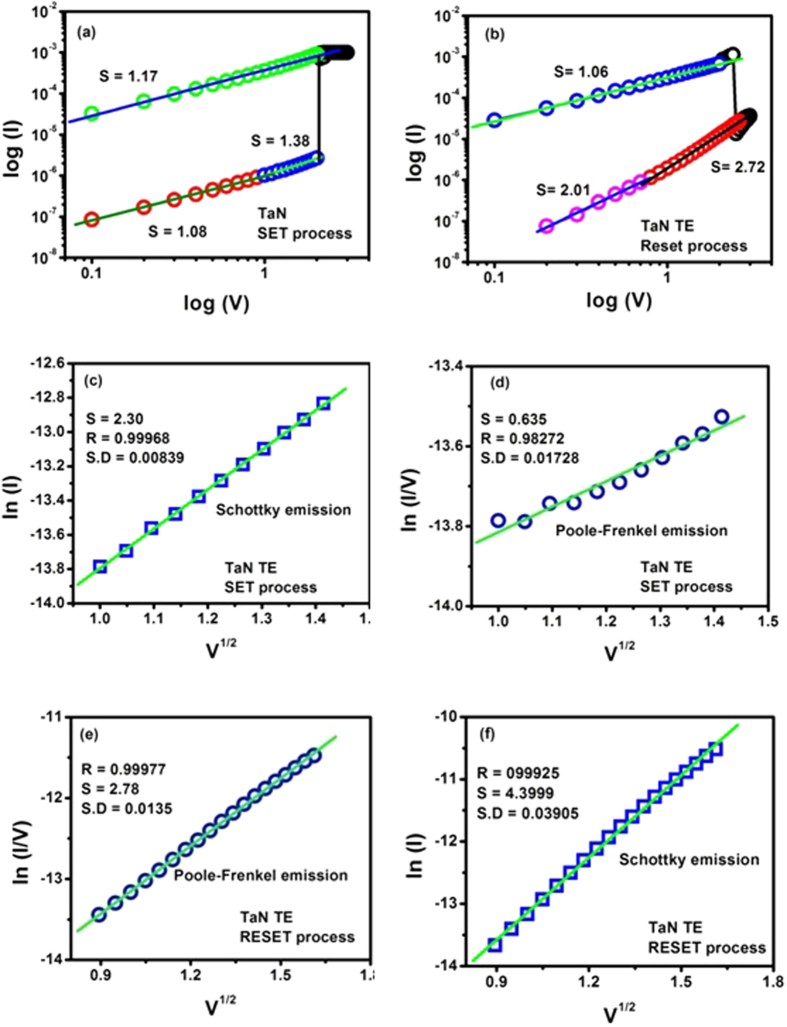
I-V curves fitted in double logarithmic scale for TaN TE memory device. (**a**) set process (**b**) reset process. I–V curves fitting by Schottky emission (**c**) and Poole–Frenkel emission (**d**) in HRS of positive bias and in HRS of higher negative bias region (**e–f**).

In order to better understand the electrical conduction behavior of Ti and TaN TE-based devices, I–V curves are fitted with theories related to different conduction mechanisms for each resistance state in both positive and negative voltage regions. Various mechanisms including Schottky emission, Poole–Frenkel, Fowler–Nordheim and space charge limited current (SCLC) behavior were attempted^47^. It was found that Ohmic conduction behavior governed the LRS due to the linear relation of ln I vs. ln V with a slope of ~ 1 for both Ti and TaN TE based devices, because the density of thermally generated free electrons in cerium oxide film is greater than the electrons vaccinated from the electrode in the positive and negative voltage region, as shown in Figs 7 and 8(a,b). At HRS, the I–V curves are nonlinear and more complicated. In low field region it is mostly ohmic except for the TaN TE base devices in the reset state where conduction obeys Child’s Law (slope ~ 2). However, in high field region, slope of lnI−lnV graphs lies in the range from 2.36 (in SET state) to 1.55 (in RESET state) for Ti TE memory devices and from 1.38 (in SET state) to 2.72 (in RESET state) for TaN TE memory devices which means that injected charge carriers are greater than bulk generated charge carriers. This might lead to the presence of traps in the cerium oxide film.

In the high field region, in order to verify whether Poole-Frenkel or Schottky emission is dominant during set process, plots of lnI (Schottky plot) and ln(I/V) (Poole-Frenkel plot) as a function of square root of applied voltage (√V) are drawn for both Ti and TaN TE based devices as illustrated in Figs 7 and 8(c,d). These curves show linear behavior for Schottky emission as compared to that for Poole-Frenkel conduction (curved plots) in the high field region (from ~ 5.0 × 10^5^ V cm^−1^ to 1.25 × 10^6^ V cm^−1^) in both Ti and TaN TE-based devices. Both Ti and TaN TE based devices demonstrate that Schottky conduction mechanism is dominant in high field region during transferring the devices from HRS to LRS (SET process). Subsequently, for RESET process same curve fitting method is adopted like that in SET process and found that both mechanisms illustrate linear relationship as obvious from Figs 7 and 8(e,f). Due to the same functional dependence of current upon voltage in both Schottky and Poole-Frenkel conduction mechanisms, the linear portion of these graphs at high field region can be taken as an evidence of either electrode-limited Schottky emission or bulk-limited Poole-Frenkel effect. This is because of the different rates of change in conductivity with field strength^48^.

As it is known that linearity in the behavior of ln (I) or ln(I/V) as a function of √V governs Schottky or Poole-Frenkel emission, respectively yielding positive gradients as under:^49^


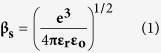



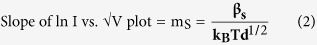



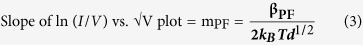


So that barrier lowering coefficients are given by:


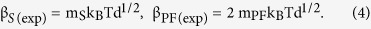


These results reveal the fact that conduction mechanism functioning at high voltage in both of these RRAM devices is Schottky emission as verified by Table 1 since the barrier height coefficient β_s_ (experimental) is approximately equal to β_s_ (theoretical). It is due to the fact that the electric field applied to the metal-insulator interface lowers the potential barrier height, so electrons ejected from the metal electrode can easily cross the potential barrier and electrode limited current flows in accordance with the Richardson-Schottky law^50^:


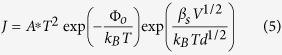


where A* is Richardson constant ~120. All other variables have their usual meanings.

As bulk of CeO_2_ film is unable to provide excess carriers, however, metallic electrode can provide increasing amount of injected electrons at high electric field in the vicinity of electrode-oxide (CeO_2_) interface. So, conduction of charge carriers controlled by top electrode confirms the Schottky mechanism.

### Analyses of DFT Calculations

**Figure 9:**
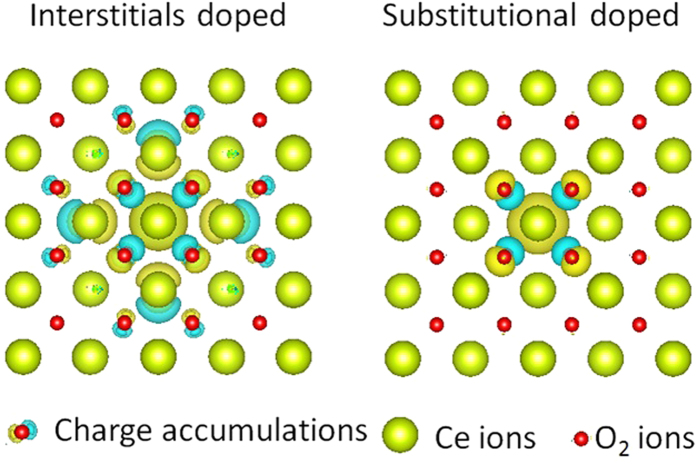
Plots of total charge density of CeO_2_ caused by the defect states; i) when Ti acts as interstitial impurity and ii) as substitutional dopant. Red balls are oxygen ions, green balls are Ce ions, while the remaining indicates charge accumulation.

To theoretically interpret the results, density functional theory was applied utilizing the VASP code. For this purpose, a 2 × 2 × 2 supercell of CeO_2_ was molded by inserting single Ti-metal atom as a dopant. Calculations of partial charge density were performed in the case of Ti atom as an interstitial as well as a substitutional impurity atom and the resulting cell structures are shown in Fig. 9. It is demonstrated that when Ti acts as an interstitial dopant in the CeO_2_ lattice, not only Ti but also oxygen atoms accumulate charges around them as clear from Fig. 9. Such accumulation of charges may consequently assist in the formation of conduction filaments and hence in the RS mechanism of interstitial systems. But on the other hand, when Ti-atom is doped as substitutional impurity (defect), it does not accumulate any charges nor generate chemical bonds with the oxygen ions as it replaces the Ce atom. Figure 9 clearly demonstrates this situation and the charge accumulation only around oxygen atoms. Present results of partial charge density calculations are well consistent with the explanation given by Zhao *et al*.^12^ and Yuanyang *et al*.^49^. They distributed the metal dopants into two groups with respect to their formation energy when these dopants are present in HfO_2_ lattice as interstitial or substitutional atoms. According to their DFT calculations, the interstitial metals can directly form conduction filaments, however, substitutional dopants are lesser favorable in directly forming CFs. Moreover, Ti belongs to Hf-like substitutional system, which contributes least in generating oxygen vacancies. Zhang *et al*.^51^ demonstrated the doping of both trivalent and tetravalent (Al and Ti) in ZrO_2_ (6.11 eV) RRAM devices. According to their findings, trivalent Al ion considerably diminished formation energy of ZrO_2_ (~2.6 eV out of 6.11 eV), while tetravalent Ti doping faintly decreased formation energy by only 0.3 eV. Such reasonable reduction in formation energy caused by Al was attributed to Coulomb interactions of dipoles formed between dopants (negatively charged acceptor) and oxygen vacancies (positively charged donors), which played vital role in improving the resistive switching characteristics as compared to tetravalent Ti-dopant^51^. Based upon all these facts, it can be concluded that Ti-dopant plays a significant role in RS behavior when it acts as an interstitial defect while as substitutional defect its performance in RS behavior is the least. Since, it provides the feeblest assistance in forming the conducting filaments^12^,^52^. That’s why, the switching process and conduction filaments formed in Ti TE- and TaN TE- memory devices are mainly due to the effect of top electrode.

### Physical Resistive Switching Mechanisms

In our previous studies, it was demonstrated that the Ti*/*CeO_2_/Al/CeO_2_*/*Pt device exhibits bipolar and unipolar RS behaviors. Resistive switching mechanism was dominated by charge transfer ability of Al dopant and oxygen vacancy generation and recombination^53^. However, in the present TaN*/*CeO_2_/Ti/CeO_2_*/*Pt devices, SET process is difficult to achieve after electroforming and RESET process occurs at a same negative voltage. As different doped impurities will cause different switching behaviors as discussed above, Ti TE devices show relatively larger SET and electroforming voltages as compared to those of TaN TE devices, indicating that some oxygen ions may attract toward TaN TE and make thinner interfacial TaON layer during SET process and the switching process will benefit from these oxygen ions.

The switching should be realized through the reversible oxidation and reduction reaction at the interface between the Ti TE and dielectric film^54^. It is worth mentioning here that Ti dopant has not extracted sufficient oxygen ions from the two CeO_2_ films to form any TiO_x_ interlayer, which is also confirmed from charge density calculations shown in Fig. 8. This indicates that if Ti is doped into CeO_2_ films interstitially, it can introduce more oxygen vacancies as compared to the insertion of Ti-ultrathin layer within the CeO_2_ films. As discussed above, the interstitial doping of Ti into CeO_2_ films can play a major role. In addition, Liu *et al*.^55^ have demonstrated that Ti doping in ZrO_2_ may act as a seed in the ZrO_2_ crystal lattice for the formation of conducting filaments. Our result seems to be consistent with the studies of Ti-doped HfO_2_ films^49^ and Ti-doped ZrO_2_ devices^55^. Present XPS results (Fig. 2) are also supporting the said fact so we believe that reduction in the SET/RESET and electroforming voltages in the TaN*/*CeO_2_/Ti/CeO_2_*/*Pt device is caused by preexisting oxygen vacancies due to formation of the TaON thinner interfacial layer. This layer migrates oxygen vacancies to form local conductive paths or supply ion transmission channels to help oxygen ions diffusion.

Work functions of top metallic electrodes such as Ti (4.33 eV) and TaN (4.61 eV) are quite close to that of dielectric CeO_2_ (4.69 eV) and so they form Ohmic like contacts at top interface of both the devices in low bias region. Moreover, oxide formation energy of Ti is noticed to be the lowest among various metals so it can readily reduce CeO_2_ and form Ohmic contact at the interface^20^,^56^. Additionally, due to the greater electronegativity of Ti (1.53 eV) than CeO_2_ (1.12 eV), Ti can be expected to attract negatively charged oxygen ions making thick layer of TiO and creating more oxygen vacancies in CeO_2_ layer in Ti TE base devices as obvious from XPS results (Fig. 2). Ti TE based devices possess lesser capability to generate new oxygen vacancies as an interlayer because of the lower enthalpy of the Ti-O compound. These oxygen vacancies are more likely to be generated around Ti ions, since the formation energies of oxygen vacancies near Ti dopants are lower, which suppresses the random formation of filaments in the film. Therefore, titanium shows a very high oxygen affinity and possibly extracts a large amount of oxygen ions from the CeO_x_ film, resulting in a high density of oxygen vacancies at the Ti/CeO_x_ top interface. As compared to Ti, TaN is lesser oxidizable by making a thin layer of TaON at top interface. Ti layer doped in CeO_2_ matrix behaves as trapping layer for electrons in RRAM devices. Under external voltage biasing, electron trapping/de-trapping phenomena operate leading to resistive switching between HRS and LRS^57^. Large interface barrier exists between Pt BE and CeO_2_ due to the larger work function difference (Pt ~ 5.69 eV and CeO_2_ ~4.69 eV) between them. The Pt metal electrodes have a deep Fermi level of ~5.7 eV which is unfavorable for the carrier injection into the conduction band of CeO_2_ with the electron affinity of about 3.5 eV^58^.

No obvious reaction related to oxidation/reduction can be expected to take place at the CeO_2_/Pt bottom interface, because Pt is electrochemically inert metal, which guarantees easy movement of the oxygen ions or atoms along grain boundaries. A Schottky barrier was thought to be established at CeO_2_/Pt bottom interface^59^. When a set voltage pulse was applied to the device, electrons injected by the Pt BE were easily trapped by the TiO/CeO_2_ top interface due to the Schottky barrier existed at the bottom interface. When all the traps filled with electrons, interface barrier decreased and a sudden current jump switched the device to LRS state. On applying negative voltage pulse, trapped electrons started to escape switching the device to HRS state again^60^,^61^. Meanwhile, the presence of abundant oxygen vacancies facilitates the operation of conducting filament formation/annihilation mechanism, which is essentially a process of vacancy drift accompanied by electrochemical redox reactions.

**Figure 10:**
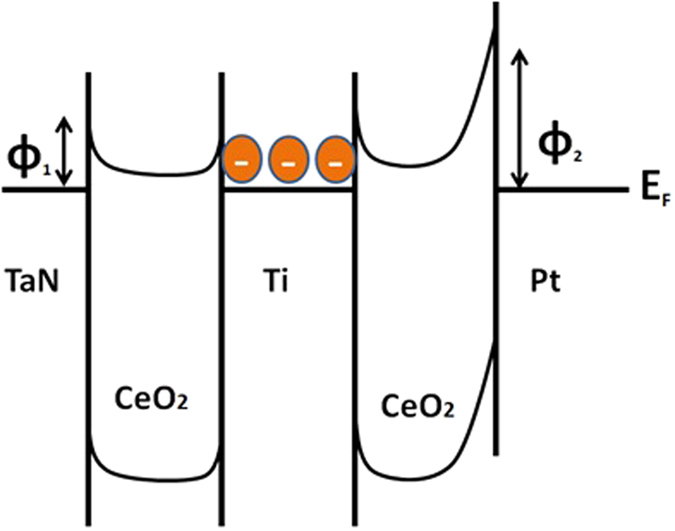
Energy band diagram of TaN/CeO_2_/Ti/CeO_2_/Pt memory stacks (work function of TaN: 4.69 eV, Ti: 4.33 eV, electron affinity of CeO_2_: 50 KJ/mole and work function of Pt: 5.6 eV).

In the case of TaN TE, when negative bias is applied to the pristine state of TE, TaON interfacial layer is formed leaving oxygen ions toward the Pt BE lowering the Schottky barrier height and completing the connecting path between top and bottom electrodes. In this way, the device is successfully switched from HRS to LRS. Based on the fact that RESET mechanism in the TaN TE device can be achieved at negative voltage, the thermal effect by Joule heating may play a major role to rupture the conducing filament. For real application, when a positive bias is applied to the TaN TE, positively charged oxygen vacancies at the interface will be repelled into the CeO_2_ layer. Due to the fact that the conductivity of ceria is sensitive to the content of oxygen element, decrease of the stack resistance should be expected. In the RESET process, a negative bias will attract the oxygen vacancies back to the interface, thus the stack returns to HRS. Moreover, the electronegativity of Pt (2.82 eV) is also much larger than electronegativity of CeO_2_ (1.12 eV) therefore lesser chances to diffuse into CeO_2_ layer^61^,^62^ as depicted in energy band diagram (Fig. 10).

## Summary

Effect of two different kinds of top electrode materials on RS performance in Ti-doped CeO_2_ thin films has been inspected. No obvious effect of Ti doping (sandwich layer) was observed on RS behavior. It was found that the TaN/CeO_2_/Ti/CeO_2_*/*Pt stack exhibited better resistive switching properties in comparison with Ti/CeO_2_/Ti/CeO_2_*/*P stack. Conduction mechanism in the TaN/CeO_2_/Ti/CeO_2_*/*Pt device is Ohmic in the low field region and in high field region charge transport is dominated by electrode-limited Schottky conduction. TaN TE memory devices exhibit low operating voltages and weak dispersion in RS parameters along with good ON/OFF ratio, excellent endurance (>10^4^ s) at RT and 85 °C and stable switching (>10000 cycles). Based on the experimental and theoretical analysis, conduction mechanism has been investigated to explain the RS behavior of both Ti and TaN TE based stacks. Formation and annihilation of oxygen vacancy based conducting filaments at thinner TaON interfacial layer has been found to play the main role in improving the resistive switching characteristics. The existence of interfacial layers between TE and CeO_2_ interfaces were confirmed by cross-sectional HRTEM images, XPS and XRD analyses. No degradation or data losses were noticed upon successive readout after performing various endurance cycles. The observed superior resistive switching behavior in TaN/CeO_2_/Ti/CeO_2_/Pt stack shows its great potential for future nonvolatile memory applications.

## Methods

In this study, the proposed resistive memory devices were fabricated as follows: A 20-nm-thick adhesion layer of Ti and a 70-nm-thick Pt film were deposited as the bottom electrode on a SiO_2_/p-Si (100) substrate using e-beam evaporation. First layer of CeO_2_ (6 nm) was deposited on Pt bottom electrode. Then an ultra-thin Ti (~1 nm) layer was deposited on CeO_2_/Pt using Ti metal target by radio frequency (RF) magnetron sputtering with 60 W power. The working pressure was maintained at 10 mTorr using 20 sccm argon gas flow. The substrate to target distance of ~4 cm was kept constant throughout the process. Deposition of ~1 nm ultra thin Ti interlayer was realized by employing much better vacuum conditions and small deposition time (in the order of 10 s). Subsequently, the second layer of CeO_2_ film (6 nm) was deposited on Ti/CeO_2_/Pt by using ceramic target of CeO_2_ (99.999% pure) via RF magnetron sputter at room temperature. Base pressure of the sputtering chamber was kept below 2.1 × 10^−6^ Torr using the turbo molecular pump. Gas mixing ratio of argon and oxygen during deposition was set to 10:20. The working pressure was kept at 10 mTorr and RF power of 100 W was applied. In this way, the three sequential layers CeO_2_*/*Ti*/*CeO_2_ were deposited by RF magnetron sputtering. Finally, 100 nm thick top electrodes (both TaN and Ti) were evaporated using electron beam evaporation, to complete the metal-oxide-Ti-oxide-metal structure to form TaN/CeO_2_/Ti/CeO_2_/Pt and Ti/CeO_2_/Ti/CeO_2_/Pt devices. Prior to top electrode deposition, top electrode was patterned as circular dots (Φ = 150 μm) using metal shadow mask. Furthermore, a protective capping layer of Pt (20 nm) was also deposited on Ti top electrode by the same method. An Agilent B1500A semiconductor parameter analyzer (Agilent Technologies, Inc., CA, USA) was used to keep track of electrical characteristics of all these CeO_2_ based memory devices. During electrical measurements, bias voltage was applied to the top electrode (Ti or TaN) while the Pt bottom electrode was kept grounded.

The formation of any interfacial layer, crystal structure and defect/oxygen vacancies concentrations in the oxide layers were examined using an X-ray diffractometer (XRD, D8, Bruker Corp.), high-resolution transmission electron microscopy (HRTEM) and X-ray photoelectron spectroscopy (XPS, ULVAC-PHI Quantera SXM). Theoretical calculations for bulk CeO_2_ dopant mechanism and electronic charge density have been done within the framework of plane-wave density functional theory (DFT) by employing Vienna ab-initio simulation package (VASP)^21^,^22^. Generalized gradient approximation along with Perdew Burke-Ernzerhof functionals and projector augmented wave (PAW) method were used to describe the interaction between ions, electrons and electron interchange effects, respectively^23^,^24^,^25^.

## Additional Information

**How to cite this article**: Rana, A. M. *et al*. Endurance and Cycle-to-cycle Uniformity Improvement in Tri-Layered CeO_2_/Ti/CeO_2_ Resistive Switching Devices by Changing Top Electrode Material. *Sci. Rep.* **7**, 39539; doi: 10.1038/srep39539 (2017).

**Publisher's note:** Springer Nature remains neutral with regard to jurisdictional claims in published maps and institutional affiliations.

## Figures and Tables

**Table 1:** Experimental values of barrier lowering coefficient **β** and Φ_o_ determined at room temperature ln I and ln (I/V) vs. V^1/2^ plots.

MIM devices	β-Theoretical (×10^−5^ eVm^1/2^V^−1/2^)		β-Experimental (×10^−5^ eVm^1/2^V^−1/2^)		β_S(exp)_/β_PF(th)_	Schottky barrier height Φ_o_ (eV)
	β_S_	β_PF_	β_S_	β_PF_		
Ti TE	**0.74**	**1.49**	**0.77**	**0.64**	**0.51**	**0.35**
TaN TE			**0.88**	**1.11**	**0.59**	**0.21**
